# Therapeutic potential of vasculogenic mimicry in urological tumors

**DOI:** 10.3389/fonc.2023.1202656

**Published:** 2023-09-21

**Authors:** Xinyu Lin, Sheng Long, Congcong Yan, Xiaofeng Zou, Guoxi Zhang, Junrong Zou, Gengqing Wu

**Affiliations:** ^1^ The First Clinical College, Gannan Medical University, Ganzhou, Jiangxi, China; ^2^ Department of Urology, The First Affiliated hospital of Gannan Medical University, Ganzhou, Jiangxi, China

**Keywords:** vasculogenic mimicry, urological, tumors, therapeutic potential, angiogenesis

## Abstract

Angiogenesis is an essential process in the growth and metastasis of cancer cells, which can be hampered by an anti-angiogenesis mechanism, thereby delaying the progression of tumors. However, the benefit of this treatment modality could be restricted, as most patients tend to develop acquired resistance during treatment. Vasculogenic mimicry (VM) is regarded as a critical alternative mechanism of tumor angiogenesis, where studies have demonstrated that patients with tumors supplemented with VM generally have a shorter survival period and a poorer prognosis. Inhibiting VM may be an effective therapeutic strategy to prevent cancer progression, which could prove helpful in impeding the limitations of lone use of anti-angiogenic therapy when performed concurrently with other anti-tumor therapies. This review summarizes the mechanism of VM signaling pathways in urological tumors, i.e., prostate cancer, clear cell renal cell carcinoma, and bladder cancer. Furthermore, it also summarizes the potential of VM as a therapeutic strategy for urological tumors.

## 1 Introduction

Urological cancers include cancers of the urinary system, including kidneys, urinary tract epithelium (including bladder, ureter, and urethra), prostate, testes, and penis. The American Cancer Society predicted in 2022 that there would be 1,918,030 new cases of cancer diagnosed with estimated 31,990 death from urological cancers in the United States. Prostate cancer is the most common malignancy and the third leading cause of cancer-related deaths in men, while kidney and bladder cancer are regarded among the top ten most common malignancies in the United States (1). The growth, invasion, and metastasis of malignant tumors are closely related to angiogenesis, which has become one of the hot topics in cancer research (2). Over the past few decades, it has been thought that the growth of tumors is dependent on angiogenesis. Tumors larger than 2mm in diameter cannot rely solely on diffusion for oxygen supply, and without the intervention of neovascularization, tumors cannot continue to grow (3). Based on this hypothesis, it has been proposed that antiangiogenic drugs should be able to inhibit the growth of all solid tumors (4). However, anti-angiogenic therapy has so far shown only limited efficacy in patients. As early as before, researchers have proposed the idea of non-angiogenic tumors. But only recently has the special biological status of non-angiogenic tumors been formally described (5). Non-angiogenic tumors grow through two main mechanisms in the absence of angiogenesis. One way is to utilize pre-existing blood vessels by infiltrating cancer cells and occupying normal tissue, this is called vessel co-option. The second is through the formation of channels that provide blood flow through the cancer cells themselves, known as vasculogenic mimicry (6). To ensure sufficient nutrient supply, tumors release angiogenic factors that promote neovascularization. The coexistence of angiogenesis and VM is common in invasive tumors, and anti-angiogenic agents have been found to have little to no effect on VM (7). The VM can replace the angiogenesis role, providing tumors with oxygen and nutrients. Further, Qu et al. reported that anti-angiogenic therapy might even facilitate the formation of VM (8). As research progresses, more and more scientists are paying attention to the potential of vasculogenic mimicry (VM) in cancer treatment, and it has been found that a treatment plan using a combination of anti-angiogenic and anti-VM drugs is imperative. Currently, VM is of particular interest in the three types of cancers mentioned above. This review will focus on the research progress made regarding VM in prostate, kidney, and bladder tumors.

## 2 Forms of tumor angiogenesis

### 2.1 Angiogenesis and vasculogenesis

Angiogenesis is the process of forming new blood vessels from pre-existing ones through sprouting, which is stimulated by endothelial growth factors promoting paracrine signaling, leading to the proliferation and migration of endothelial cells, in addition to recruitment of other cell types such as smooth muscle cells (9). Angiogenesis occurs under physiological conditions, such as during embryonic development or adult wound healing. However, half a century ago, Dr. Judah Folkman proposed that pathological angiogenesis is mandatory for the growth of solid tumors (10). Cancer is characterized by the dysregulated function of angiogenesis, where the newly formed blood vessels no longer regulated by the body will promote tumor growth, metastasis, and invasion (11). The classical angiogenesis theory believes that there are two modes of tumor angiogenesis: angiogenesis based on the original blood vessel (12). The other is vasculogenesis (13). At present, more research is angiogenesis, that is, host mature vascular endothelial cells are exposed to pro-angiogenic factors in the tumor microenvironment, such as vascular endothelial growth factor (VEGF), basic fibroblast growth factor (bFGF), platelet-derived growth factor (PDGF). Under the action of these angiogenic factors and chemokines, a new collateral blood vessel of the tumor tissue is formed to provide nutrients to the tumor tissue (14). Another form of tumor angiogenesis is vasculogenesis, which refers to endothelial precursors derived from bone marrow and hematopoietic cells, called endothelial progenitor cells (EPCs) (15). And through growth factors, cytokines and hypoxia-related signaling pathways recruited to the tumor site, where they differentiate into mature endothelial cells, and under the stimulation of angiogrowth factor, division, proliferation, endothelial cells clump together to form a vascular channel to supply nutrients to tumor cells (16, 17). Targeting tumor angiogenesis has become an important target in cancer treatment, as angiogenesis plays a significant role in tumor progression. Targeted drugs such as bevacizumab, Sorafenib, and Sunitinib have been successfully used in clinical practice, advocating the success of anti-angiogenic therapy in cancer treatment (18). However, some studies have reported that these drugs have poor therapeutic effects on certain patients and could even promote tumor progression (19, 20). Therefore, some researchers speculated that new microcirculation patterns might exist for tumor blood nutrition supply.

### 2.2 Vessel co-option

**Figure 1:**
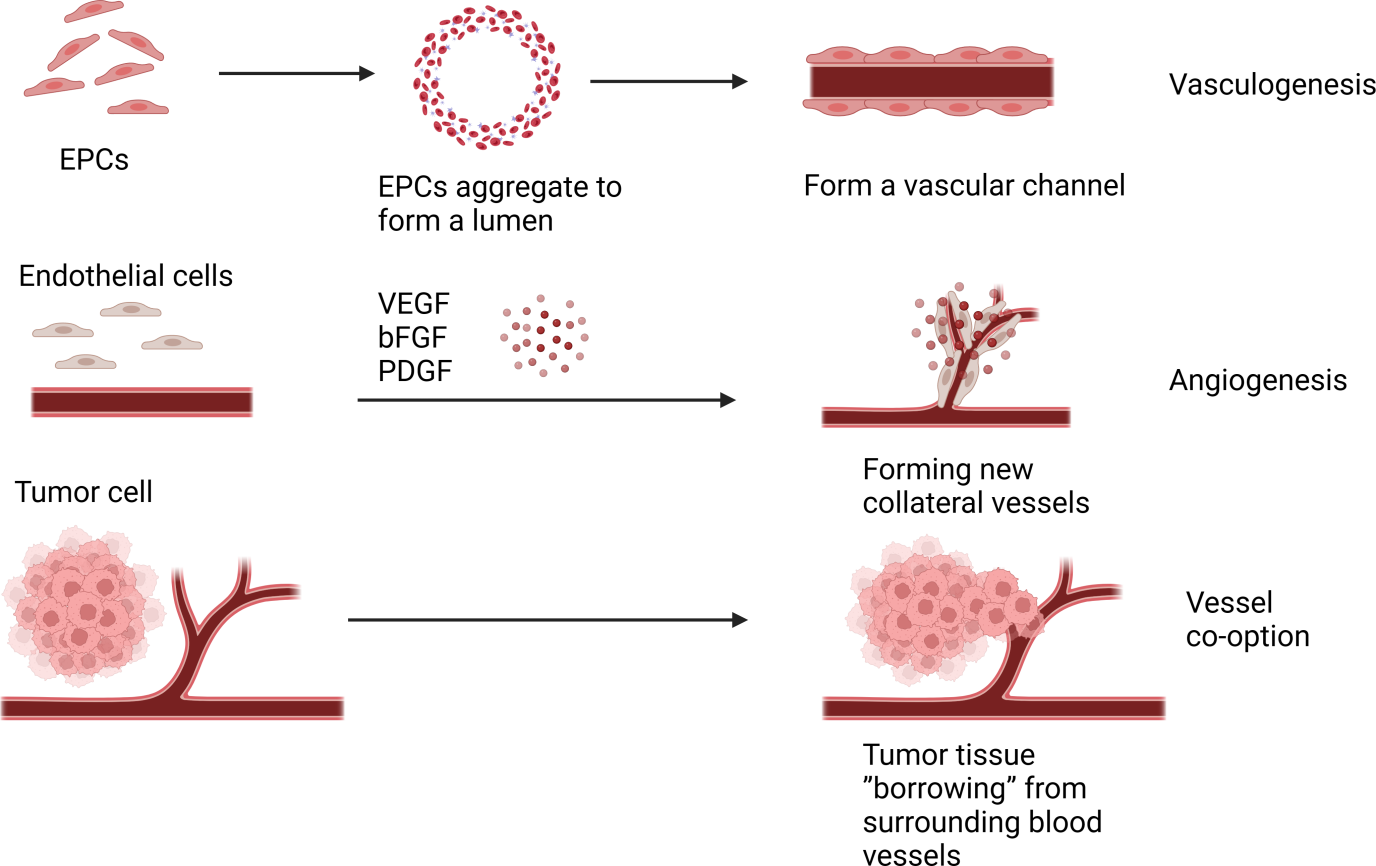
Molecular mechanisms related to VM in urological tumors. This figure summarizes the signal pathways involved in VM regulation in urological tumors. Red represents VM related signaling pathway in prostate cancer, yellow represents VM related signaling pathway in renal cell carcinoma, and the green represents VM related signaling pathway in bladder cancer.

Vessel co-option(VCO) is a phenomenon associated with tumor growth and progression, which differs from the traditional tumor angiogenic process. In tumor co-selection, tumor cells do not induce new angiogenesis, but choose to “borrow” existing blood vessels from surrounding normal tissues to supply their own nutritional and oxygen needs (6). In some cases, cells from malignant tumors move along existing vascular pathways, invading and occupying the blood vessels of normal tissue, thereby obtaining nutrients and oxygen from the blood (21). VCO causes less prominent vascular structures in morphology, making tumors more difficult to detect (22). VCO has been observed in a variety of cancers, such as liver, brain, skin, lymph node, and many others (23–25). Several studies have shown that many solid tumors can progress through vascular co-selection, and blocking co-selection and anti-angiogenic therapy can more effectively inhibit tumor growth (26–28). And the effect of inhibiting VCO can be achieved by targeting the signaling pathways related to VCO, such as targeting the Ang-2 pathway, VEGF pathway and YAP-TAZ pathway (29). Therefore, understanding tumor co-selection is essential to develop more precise treatment strategies and predict tumor growth patterns (**Figure 1**).

### 2.3 Vasculogenic mimicry

In 1999, Maniotis et al. discovered the formation of tumor blood vessels lacking endothelial cells in highly invasive melanoma tissue samples. These vessels were positive for PAS staining, but staining of endothelial cell markers (Factor VIII-related antigen and CD31) failed to stain the luminal contents of the vessels (30). Similarly, culturing highly invasive cell lines in a 3D extracellular matrix (ECM) demonstrated that tumor cells cultured *in vitro* produce patterned vascular channels and are PAS-positive, indicating its functionality as a blood vessel. This phenomenon was named VM (30). With further research, VM was also reported in other invasive tumors such as breast, ovarian and liver cancer and some urological tumors (31–35). VM is closely related to tumor growth, invasion, metastasis, and patient prognosis, and patients with VM generally had a shorter survival time and poorer prognosis (36).

Current research suggests that VM formation may be primarily related to cancer stem cells (CSCs) and epithelial-mesenchymal transition (EMT). CSCs are a subset of tumor cells with self-renewal and differentiation capabilities and are considered the main drivers of tumor growth, metastasis, and recurrence (37). Among them, the VEGF (vascular endothelial growth factor) pathway is considered the most important, promoting the generation of new blood vessels through signal transduction mediated by VEGFR. Mirshahi et al. found that CD133+/CD34+ stem cells derived from acute leukemia (AL) patients could secrete more IGF-1 and SDF-1, leading to the formation of VM in Matrigel (38). In melanoma, Lai et al. found that a population of cells with stem cell-like characteristics, marked by CD133, drove tumor growth by promoting VM formation and the morphogenesis of a specialized perivascular niche (39).

**Figure 2:**
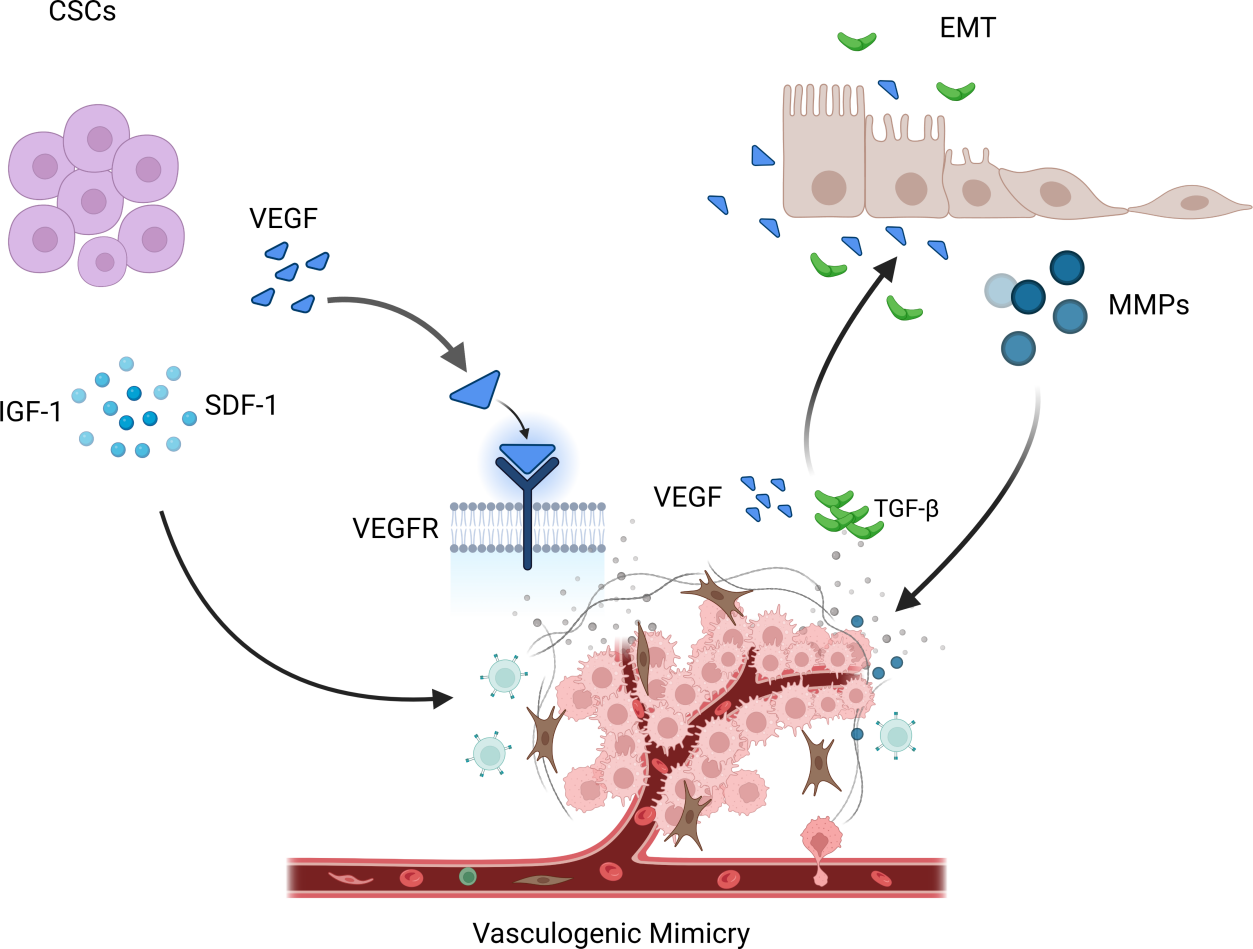
Angiogenesis is under the action of these angiogenic factors and chemokines, a new collateral blood vessel of the tumor tissue is formed to provide nutrients to the tumor tissue. Vasculogenesis is endothelial cells clump together to form a vascular channel to supply nutrients to tumor cells. Vessel co-option(VCO) is the tumor cell choose to “borrow” existing blood vessels from surrounding normal tissues to supply their own nutritional and oxygen needs.

EMT is the process by which epithelial cells transform into mesenchymal cells and plays a vital role in embryonic development, inflammation, fibrosis, and cancer progression (40). It has been reported that EMT activation triggers cancer cell invasion and metastasis and contributes to VM (41). During EMT, epithelial cells lose polarity and epithelial characteristics and acquire mesenchymal cell features (13). These cells can attract endothelial cells by releasing various cytokines and signaling molecules, promoting endothelial cell migration and invasion, thereby promoting angiogenesis and expansion of the vascular network. In addition, EMT also releases some matrix-degrading enzymes, such as MMPs, which can degrade the matrix and provide more space for new blood vessels (42). On the other hand, tumor vasculature can also inversely affect the process of EMT. Newly formed tumor vessels can release various factors such as VEGF and TGF-β (transforming growth factor-β), promoting EMT of tumor cells, thereby enhancing their invasive and metastatic ability (43–45). In addition, the lack of tumor vasculature can lead to tumor cell hypoxia, thereby promoting the occurrence and progression of EMT (**Figure 2**).

Moreover, some studies have suggested an interaction between tumor angiogenesis and immune evasion. Vasculogenic mimicry can weaken the body’s immune system’s attack on tumors by forming immune escape areas on the vascular wall (46). Therefore, simultaneously inhibiting tumor vasculogenic mimicry and enhancing the immune system’s ability to attack tumors may be an important therapeutic strategy in treating tumors.

## 3 Factors involved in urological tumor vasculogenic mimicry

### 3.1 Vasculogenic mimicry in prostate cancer

Prostate cancer (PCa) is one of the most common solid malignancies in the male urogenital system, second only to lung cancer in incidence, and ranked second among male malignant tumors (47). The treatment for localized PCa includes radical surgery and radiation therapy, while androgen deprivation therapy (ADT) is used for metastatic PCa. However, after 18 months, most metastatic PCa eventually progresses to ADT-resistant PCa (48). In patients with PCa, the incidence of VM is higher in those with higher Gleason scores, TNM staging, more lymph nodes, and distant metastases (32).

The presence of VM in prostate cancer is associated with higher expression of certain related factors, like HIF1α, EphA2, ZEB1, and Sp1. Luo et al. found that MCT1 can stabilize HIF1α through lactylation by introducing MCT1 into PCa cells, thereby promoting the transcriptional activity of KIAA1199 (49). KIAA1199 further reduces the expression of Sema3A, increases the expression of VE-cadherin and phosphorylated EphA2, and enhances angiogenesis and vasculogenic mimicry in prostate cancer by enhancing hyaluronic acid-mediated VEGFA signaling (49). EphA2 is a receptor tyrosine kinase expressed in most epithelial cells (50). In gastric cancer cells, cancer-associated fibroblasts overexpressing EphA2 promote VM formation by activating the EphA2-PI3K pathway (51). While PI3K is a heterodimeric protein composed of a catalytic subunit (p110α/β/γ/δ) and a regulatory subunit (p85α/β) (52). Wang et al. demonstrated that higher levels of EphA2 expression and PI3K activity were associated with VM in more invasive prostate cancer cell lines PC3 and DU-145, but with no significant correlation between EphA2 and PI3K expression levels (53), Luo et al. suggested that PI3K is necessary for VM in PCa and may function by regulating the phosphorylation of EphA2 (53).

*ZEB1* is a critical activator of EMT, which upregulates tumor cell plasticity and EMT to acquire cancer stem cell properties (54). In a previous study, Peng et al. found that *ZEB1* promotes EMT in lung cancer cells by activating Fak/Src signaling (55). Wang et al. found that *ZEB1* is required for VM formation *in vitro*, mediating the expression of EMT-related and CSC-related proteins in PCa cells.The study data showed that ZEB1 knockdown reduced the inhibition of Src phosphorylation at the p-Src527 site in PCa cells while reducing the formation of VMs. They further confirmed that treating PCa cells with the Src inhibitor PP2 resulted in a decrease in VM formation, while Src overexpression in stable ZEB1 knockdown cells restored VM formation (56). Where similar results were also observed in *in vivo* studies, indicating depletion of *ZEB1* protein in PC3 cells inhibited the growth of xenograft tumors in mice (56).

The transcription factor Sp1 is overexpressed in many types of cancer cells, including PCa, and is associated with various fundamental biological processes. It has been shown to play an important role in cell growth, differentiation, apoptosis, and carcinogenesis (57). Han et al. found that Sp1 controls the nuclear expression of the transcription factor twist to regulate the expression of VE-cadherin in PC3 cells. Sp1 induces the upregulation of twist/VE-cadherin, activating the AKT pathway, activated AKT enhances the expression of matrix metalloproteinases (MMPs) such as MMP-2 and -14, leading to VM formation by remodeling the extracellular matrix including LAMC2, leading to VM occurrence and development (58). Sp1, Twist, VE-cadherin, and AKT form a loop, and targeting Sp1 expression may provide a new therapeutic strategy for PCa patients with VM.

As mentioned earlier, VM formation involves proliferation, migration, and invasive changes. Therefore, the treatment for inhibiting VM can target any of these processes. Kaempferol is a natural flavonol found in many fruits and vegetables, which was reported to significantly inhibit the proliferation of AR-positive prostate cancer cell line LNCaP and promote apoptosis (59). Resveratrol (3,5,4’-trihydroxy-trans-stilbene, RES) is one of the most well-known phytochemicals found in red wine, grapes, berries, and peanuts with potent antioxidant and anticancerous properties (60). Han et al. found that RES inhibited the VM structure formation at non-cytotoxic concentrations by inactivating EphA2 and reducing twist-mediated VE-cadherin expression when co-cultured with prostate cancer cell line PC-3 cells (61). Chrysin, also known as 5,7-dihydroxyflavone, is another natural compound with anti-tumor properties, chrysin inhibits the growth and VM formation of prostate cancer cell line PC-3 by inhibiting HIF-1α, SPHK-1, and phosphorylation of the AKT/GSK-3β signaling pathway (62).

### 3.2 Vasculogenic mimicry in renal cell carcinoma

Renal cell carcinoma (RCC) accounts for 3.8% of all cancers and 2.5% of all cancer deaths worldwide (63). Early-stage RCC is commonly treated with partial or radical nephrectomy, with a 5-year survival rate of 92.6%. However, about 25% of RCC patients are diagnosed at the metastatic stage and one-third of patients undergoing local tumor resection experience recurrence (64). Angiogenesis is a key aspect of pathogenesis, and anti-angiogenic drugs such as Sunitinib have been shown to significantly reduce tumor blood flow. However, most Sunitinib-treated patients eventually experience tumor progression after several months of treatment (65). More than a decade ago, Amalia A Vartanian et al. found through retrospective studies that RCC patients who tested positive for VM had a significantly lower disease-free survival rate and a significantly increased risk of recurrence (66). Therefore, VM as a novel neovascularization pathway cannot be ignored in treating RCC.

The MMP family plays a role in promoting VM progression in various cancers. Lin et al. found that MMP9 was overexpressed in RCC patient tissues, which was positively correlated with clinical stage, pathological grade, RCC metastasis, and VM formation (67). Targeted inhibition of MMP9 prevented the formation of VM in RCC cell lines 786-O and 769-P that were originally able to form VM (67). *Vimentin* (*VIM*) is a major component of the intermediate filament (IF) protein family and a hallmark of EMT (68). In RCC, *VIM* overexpression is one of the independent predictors of poor clinical outcomes (69). Many studies have also shown that *VIM* plays an important role in the formation of VM (67, 69–71). Bai et al. found that TR4 downregulates the expression of miR490-3p, which upregulates *VIM* expression, thereby promoting RCC VM formation and metastasis (72). Lin et al. further validated the role of *VIM* in promoting VM in RCC by inducing EMT through hypoxia, upregulating *VIM* and *AXL*, and downregulating E-cadherin expression to promote RCC cell VM formation (71). He et al. found Sunitinib regulates ERβ signaling to increase cancer stem cell and angiogenic mimicry formation (73), However, first-line anti-angiogenesis drugs such as Sunitinib or Bevacizumab cannot inhibit VM and may even induce VM, Ding et al. found that targeting ERβ/circDGKD by downregulating VE-cadherin reduced RCC growth and proliferation and significantly weakened the VM formation, which is envisaged to enhance the efficacy of Sunitinib providing a new combinational therapy strategy to prevent RCC progression (74).

Paired-related homeobox 1 (PRRX1) is a novel inducer of EMT, and its expression is associated with metastasis and prognosis in multiple tumors (75, 76). Protein phosphatase 2A (PP2A) is an effective tumor suppressor that acts on various oncogenic transcription factors (77). CIP2A is an important oncogene that inhibits the activity of PP2A, thereby maintaining the malignant phenotype of tumor cells and playing an important role in the occurrence, development, and biological behavior of tumor cells (78). Both PRRX1 and CIP2A are major inducers of EMT. Wang et al. investigated the roles of PRRX1, CIP2A, and VM in RCC and found that PRRX1 expression was negatively correlated with VM and CIP2A, whereas CIP2A expression was positively correlated with VM development. Low PRRX1 expression combined with high VM and CIP2A was associated with poor prognosis and metastasis in RCC (79). However, Wang et al. only observed this correlation in RCC patient specimens and did not perform animal or cell line investigations to explore the underlying mechanisms. Further research is needed to clarify the mechanisms involved.

Androgen receptor (AR) has an oncogenic function in RCC, promoting progression and hematogenous metastasis (80). You et al. showed that a long non-coding RNA, lncRNA-TANAR, regulated by AR transcription, increased the stability of TWIST1 mRNA by directly binding to its 5′UTR, disrupting UPF1-mediated nonsense-mediated TWIST1 mRNA decay, thereby leading to a decrease in VM formation (81).

Tumor-associated macrophages (TAMs) play a crucial role in reshaping the tumor microenvironment (TME) to promote tumor development (82). Numerous studies have shown that TAMs can promote tumor cell proliferation, invasion, and migration (83, 84). Polarized macrophages commonly exist as either M1 or M2 macrophages. Unlike M1 macrophages, which have pro-inflammatory and immune-stimulatory effects, M2 polarized macrophages are similar to TAMs and have pro-tumor functions (85). It has been found that macrophages can affect cancer progression through miRNAs carried by extracellular vesicles (86). Liu et al. evaluated ten VM-related genes in RCC cells co-cultured with or without TAMs using protein imprinting and found that TIMP2, which was restrained by TAMs, might be a key VM regulatory factor in RCC (87). Subsequently, through bioinformatics analysis and experimental validation, Liu et al. found that miR-193a-5p derived from macrophage-derived extracellular vesicles targeted TIMP2 in RCC cells, enhancing VM and cell invasion capability (87).

Metabolic reprogramming is a hallmark of cancer and is critical in tumor progression. Accumulation of the tumor metabolite L-2-hydroxyglutarate (L-2HG) occurs in cancer due to hypoxia (88), which is also an important factor in VM formation. Wang et al. found that tumors with high levels of L-2HG exhibited more VM structures than tumors with low levels of L-2HG. They also compared RNA sequencing analysis of RCC cell lines with and without L-2HG treatment and found that PHLDB2 was downregulated by L-2H (89). PHLDB2 (also known as LL5β) is a protein containing a PH domain that plays an important role in mediating cell migration by forming complexes with partners such as CLASPs and Prickle 1 (90). Wang et al. found that inhibiting PHLDB2 reduced VM formation while restoring PHLDB2 expression levels reversed this phenomenon. They also found that decreasing PHLDB2 expression increased ERK1/2 phosphorylation, but there was no statistically significant difference in ERK1/2 phosphorylation due to limited replicates. However, the trend of changes in ERK1/2 phosphorylation was consistent in each group (89). Therefore, the L-2HG/PHLDB2 pathway may be a potential signaling pathway for treating VM in RCC.

### 3.3 Vasculogenic mimicry in bladder cancer

It is estimated that there are reported 500,000 new cases and 200,000 deaths from bladder cancer (BCa) globally. Over 80,000 new cases are reported in the United States alone, with 17,000 deaths from BCa annually (91). Despite various treatments such as surgery, bladder infusion, and immunotherapy being used in clinical practice, the rate of tumor progression within 5 years is still very high (92). In particular, the treatment options for advanced BCa are minimal (93). Early on, Fujimoto et al. found that ECV304, derived initially from BCa epithelial cells and now known as the T24/83 BCa epithelial cell line, can connect with blood vessels around the normal endothelial source, forming tumor tissues with vascular characteristics and is typically found in highly invasive tumors with poor prognosis (94).

A protein TG2, which has been demonstrated to be associated with endothelial cell-derived angiogenesis (95), can be overexpressed under pathological and stress conditions, leading to increased cell surface externalization and deposition into the extracellular matrix (ECM), thereby exerting crosslinking effects with various ECM proteins such as fibronectin and laminin, etc. (96). Moreover, previous studies have suggested that exogenous TG2 added to a rat dorsal skin flap wound healing model can enhance angiogenesis (97). Jones et al. found that TG2 was not detected in normal human fibroblast C378, while there was an abundant expression of TG2 in ECV304 cells. Targeted inhibition of TG2 expression in ECV304 cells could block cell migration, thereby preventing the formation of the actin cytoskeleton and focal adhesion (98).

MicroRNAs (miRNAs) are evolutionarily conserved small non-coding RNAs that function as endogenous regulators of gene expression (99). Dysregulation of certain miRNAs has been associated with numerous tumorigenic changes, including growth, apoptosis, metastasis, and tumor angiogenesis (100). It has been reported that miR-124 inhibits the malignant potential, proliferation, and invasiveness of malignant tumor cells by targeting multiple proteins (101–103). Studies have shown that *UHRF1* is an oncogene promoting cancer cell development (104). In different types of cancers, the expression of *UHRF1* incurs many changes getting out of control. The expression or activity of this protein is often modified, leading to transformation and increased proliferation, motility, and invasiveness, as well as providing tumor cells with resistance to chemotherapy (105). Wang et al. found that miR-124 and *UHRF1* are negatively correlated in BCa tissue, where miR-124 inhibited BCa invasiveness by reducing *UHRF1* expression (106).

Many studies have proposed that EMT is crucial for VM formation and tumor progression, with *ZEB1* as an essential EMT inducer that is elevated in colorectal cancer specimens showing EMT features both *in vivo* and *in vitro *(107). Li et al. found that *ZEB1* is also highly expressed in BCa, and to further elucidate the relationship between VM and *ZEB1* in BCa, they performed 3D culture assays after transfection with specific siRNA to reduce *ZEB1* expression in bladder transitional cell carcinoma cell lines. Moreover, after *ZEB1* restoration, VM formation was inhibited in UM-UC-3 and J82 cell lines (35). However, Li et al. did not observe changes in EMT markers after suppressing *ZEB1* expression in BCa, suggesting that *ZEB1* is an intermediate step in BCa VM formation, regulated or influenced by some unknown upstream molecules and downstream genes, and it may not have a direct relationship with epithelial phenotype (35).

It is well known that the tumor microenvironment exists in a hypoxic state (108). In the hypoxic microenvironment, tumor cells form new blood vessels to obtain the oxygen and nutrients they need to support their continued proliferation. Numerous studies have shown that hypoxia is closely related to the development of VM. For example, in the melanoma mouse model, mice in the ischemic model group were found to exhibit higher VMs compared to the control group, which was positively correlated with HIF-1α and HIF-2α expression, indicating that hypoxia promoted VM (109). Liu et al. developed and validated a novel hypoxia risk score that can predict clinical outcomes and TME characteristics of BLCA, and for patients in the high-risk score group, they may benefit from immunotherapy, chemotherapy, and radiotherapy, and patients in the low-risk score group may benefit from targeted therapy with VM-associated signaling pathways (WNT-β-catenin network, PPARG network, and FGFR3 network), contributing to the development of BCa precision medicine (108).

In recent years, there has been increasing evidence that the status and formation of angiogenic mimetics (VMs) in the tumor microenvironment is regulated by various factors, especially the immune factors present in the tumor microenvironment (110). BCATRANSFERASE 2 (BCAT2) is a core enzyme in sulfur amino acid metabolism (111). Cai et al. found that BCAT2 has the effect of modulating TME immune status, and patients with high BCAT2 expression may have better efficacy in anti-VM therapy (112). Siglec15, a member of the sialic acid-bound immunoglobulin-like lectin family, is an emerging broad-spectrum target for normalizing cancer immunotherapy (113). Jiao et al. found that BCa patients in the high Siglec15 group were more sensitive to targeting vascular mimicry-related signaling pathways (β-catenin, PPAR-γ, and FGFR3 pathways) (114, 115). Therefore, Siglec15 may be used as an indicator of targeted therapy for VM.

In BCa, DNA methylation plays a key role in early diagnosis, predicting prognosis, predicting therapeutic opportunities, and serving as a potential therapeutic target (116), 5-Methylcytosine (5mC) in DNA is the most important epigenetic modification that shapes TME by influencing genomic stability, determining cancer cell differentiation status, and selecting cell identity (117, 118). Jiao et al. found that the high 5 mC score group may not be sensitive to neoadjuvant chemotherapy, but there are several immunosuppressive oncogenic pathways significantly enriched in the heterogeneous high 5 mC score group associated with VM, including WNT-β-catenin network, FGFR3 network, and VEGFA (114, 119–121). These VM pathways may provide promising therapeutic opportunities for BLCA patients in the high 5mC scoring group.

**Figure 3:**
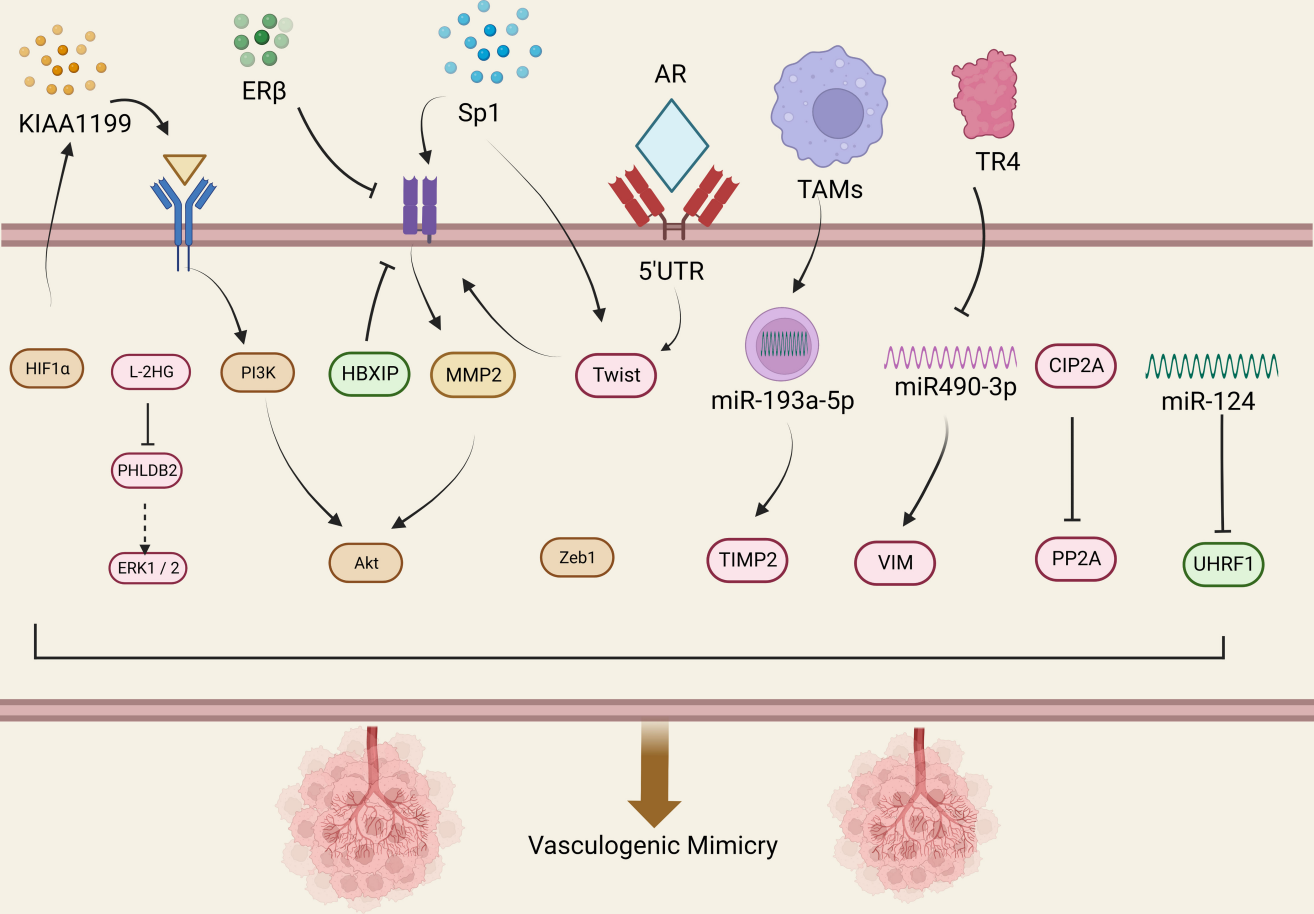
VM is regulated by various mechanisms. CSCs promote the occurrence of VM phenomenon by releasing IGF-1, SDF-1, and VEGF. During EMT, some matrix degrading enzymes are released, such as MMPs, which can promote the occurrence of the VM phenomenon. At the same time, new tumor blood vessels release various factors, such as VEGF and TGF- β, thereby promoting the occurrence and progress of EMT.

The utilization of glucose in tumor cells significantly increases, producing a large number of intermediate metabolites through glycolysis to meet the needs of tumor cell proliferation. Increasing evidence indicated that accumulated lactate, as the final product of glycolysis, is a key regulatory factor in tumor development, immune escape, metastasis, and angiogenesis (122, 123). Hepatitis B X-interacting protein (HBXIP), also known as LAMTOR5, is a conserved protein that is often expressed in various tissues in mammals (124). HBXIP is highly expressed in several types of cancers and is associated with a series of clinical pathological features and poor prognosis (125). Overexpression of HBXIP in BCa tissues is related to clinical staging, lymph node metastasis, tumor recurrence, and patient survival. In addition, silencing HBXIP reduces the proliferation, migration, and invasion of BCa cells *in vitro* and tumor formation *in vivo *(126). Moreover, the high expression of HBXIP in high-grade tissues suggests that HBXIP may be an important indicator for judging the prognosis of BCa patients. Some studies have shown that the PI3K/AKT/mTOR pathway is a central signaling pathway that coordinates aerobic glycolysis and cell biosynthesis in malignant tumor cells (127). Liu et al. provided evidence that HBXIP as an oncogene regulates glycolysis of BC cells through the AKT/mTOR pathway, thereby promoting VM in BCa cells (128). They found that reducing HBXIP in BCa cells affected the migration and angiogenesis of HUVECs and decreased the expression of VEGF and EPO. Both glucose and lactate stimulation reversed the cell viability, migration, and tubular formation of HUVECs co-cultured with HBXIP-silenced BCa cells. Glucose stimulation demonstrated that HBXIP further promotes glycolysis by regulating glucose uptake by tumor cells, while lactate stimulation demonstrated that glycolysis further promotes VM, suggesting HBXIP plays a key role in VM (**Figure 3**) (128) (**Table 1**).

**Table 1:** Genes and their functions related to vascular mimicry in urological tumors.

Cancer Type	Factors	Signaling pathway	Function	Ref.
Prostate cancer	KIAA1199	EphA2-PI3K pathway	reduces the expression of Sema3A, increases the expression of VE-cadherin and phosphorylated EphA2	(49)
	ZEB1	Fak-Src pathway	a critical activator of EMT, upregulates tumor cell plasticity and EMT to acquire cancer stem cell properties	(55, 56)
	Sp1	Twist-VE-cadherin pathway	induces the upregulation of twist/VE-cadherin	(58)
Renal cell carcinoma	TR4	miR490-3p/vimentin signals pathway	downregulates the expression of miR490-3p	(72)
	VE-cadherin	ERβ/circDGKD pathway	reduced RCC growth and proliferation and significantly weakened the VM formation	(74)
	PRRX1		a novel inducer of EMT	(76)
	PP2A		an effective tumor suppressor that acts on various oncogenic transcription factors	(77)
	CIP2A		inhibits the activity of PP2A	(78)
	AR		increased the stability of TWIST1 mRNA by directly binding to its 5′UTR	(81)
	L-2HG	L-2HG/PHLDB2 pathway	downregulated PHLDB2	(89)
	PHLDB2	L-2HG/PHLDB2 pathway	mediating cell migration	(89)
Bladder cancer	TG2		increased cell surface externalization and deposition into the extracellular matrix (ECM)	(96)
	miR-124		miR-124 inhibited BCa invasiveness by reducing *UHRF1* expression	(106)
	BCAT2		the effect of modulating TME immune status	(112)
	Siglec15	β-catenin, PPAR-γ, FGFR3 pathways	an emerging broad-spectrum target for normalizing cancer	(113)
	HBXIP	PI3K/AKT/mTOR pathway	promotes glycolysis by regulating glucose uptake by tumor cells	(128)

## 4 Potential targeted drugs for vasculogenic mimicry

### 4.1 Targeting VEGF and VEGFR

It is now widely believed that VM plays a crucial role in tumor growth, proliferation, and metastasis formation. VEGF is one of the important promoting factors for VM, and the VEGF and its receptor are one of the main inducers of tumor angiogenesis (129). Inhibitors targeting the VEGF/VEGFR system have been used clinically. Clinical trials using VEGF molecules can induce moderate improvement in overall survival, measured in weeks to just a few months, and tumors respond differently to these drugs (130). Antibodies targeting VEGF, such as Bevacizumab, can effectively inhibit tumor angiogenesis and growth and have been widely used to treat various cancers (130). In addition to directly inhibiting VEGF, tumor angiogenesis can also be inhibited by hindering the activity of VEGF receptors. Sorafenib and Sunitinib are tyrosine kinase inhibitors that block VEGFR-2, which are currently approved for treating cancers such as hepatocellular carcinoma, neuroendocrine pancreatic tumors, and metastatic renal cell carcinoma (130, 131). However, tumor cells are prone to develop resistance to these drugs, resulting in poor clinical efficacy (132).

Studies have shown that when VEGF is blocked, other angiogenic factors modulate sensitivity against VEGF therapy and allow regeneration of the tumor-associated vasculature (133). A phase III trial by Rini et al. on bevacizumab plus interferon α in patients with metastatic renal cell carcinoma showed that bevacizumab blocked the VEGF pathway and that patients with bevacizumab plus IFN-α had significantly improved PFS and OS compared with IFN-α alone (134). Although the data emphasize the importance of VEGF signaling, there are many intracellular pathways in tumorigenesis VM, and inhibitors targeting individual signaling pathways have limited inhibitory effect on VMs because other signaling pathways immediately compensate and eventually resume the process of switching to the VM phenotype (135). Therefore, exploring multi-target combination therapies to limit VM-mediated tumor resistance is expected to maximize anti-tumor efficacy in the future.

### 4.2 Targeting extracellular matrix

An extracellular matrix (ECM) is a complex extracellular biological macromolecular network involved in angiogenesis. Drugs targeting ECM can inhibit angiogenesis by interfering with the biological functions of ECM. MMPs is particularly important in ECM degradation. Under hypoxic conditions, high expression of MMP-9 molecules increases tumor invasiveness and promotes VM (136). Therefore, MMP inhibitors can inhibit MMP activity, thus blocking ECM degradation and remodeling and reducing VM. In addition, ECM is an important structure for cell adhesion, and inhibiting the binding of ECM to cells can inhibit the proliferation and migration of endothelial cells. For example, using RGD peptide sequences can block the binding of ECM to cells, thereby inhibiting VM (137).

### 4.3 Targeting PI3K/Akt/mTOR

The PI3K/Akt/mTOR signaling pathway is a crucial pathway in regulating cell proliferation, survival, and metabolism and is considered an important regulatory mechanism for tumor angiogenesis and development (138). Drugs targeting this pathway, such as Rapamycin, have been extensively studied to inhibit tumor angiogenesis and growth, thereby suppressing tumor progression and metastasis (139). Huang et al. found that under normal oxygen or hypoxic conditions, with the increase of rapamycin concentration, the duct-forming structure of glioma cell line U87-MG in stromal gum decreased, demonstrating that inhibition of mTOR can eliminate glioma VM formation (139). Therefore, Huang et al. deduced that the mTOR signaling pathway is related to VM formation and that mTOR is an upstream molecule of HIF-1α. In the final stage of the VM signaling pathway, express and activate MMP-14 to activate MMP-2. MMP-2 combined with MMP-14 cuts Ln-5-γ2 chains into migration fragments. The release of fragments of these into the tumor microenvironment can increase the migration of tumor cells, invasion, and eventually lead to VM (139). It is worth mentioning that since PI3K/Akt/mTOR signaling pathway also plays an important physiological role in normal cells, these drugs lack selective activity on cancerous cells and thus may adversely affect the normal cells (140). The results of a recent study have shown a delicate balance between the growth-promoting activity of AKT and the growth-promoting activity of p53, which is essential for preventing cellular aging and cancer (141). PI3K/AKT/mTOR inhibition also has associated clinical adverse effects, including hyperglycemia, hyperlipidemia, myelosuppression, and severe hepatotoxicity (142). Further research and optimization of targeted therapy for the PI3K/Akt/mTOR signaling pathway are imperative to improve treatment efficacy and reduce the incidence of adverse reactions.

### 4.4 Targeting perivascular cells

The tumor microenvironment contains a series of non-cancer cells around the tumor, such as fibroblasts, macrophages, and endothelial cells, as well as some ECM and molecular signaling substances. These non-cancerous cells and molecules play an important role in tumorigenesis, growth, invasion, metastasis, and tumor angiogenesis (143). In tumor angiogenesis, tumor cells release some promoting factors, such as VEGF, PDGF, etc., to stimulate tumor cells to generate VM. At the same time, tumor cells induce surrounding fibroblasts and macrophages to transform into tumor-related cell types, releasing various factors that promote tumor growth and invasion (144). Therefore, targeting non-cancer cells and molecules in the tumor microenvironment has become a strategy for treating tumor angiogenesis. Among them, drugs targeting fibroblasts and macrophages, such as Imatinib and Dasatinib.Studies have shown that dasatinib can effectively inhibit the growth of fibroblasts by inhibiting PDGF receptor signaling at biologically relevant concentrations (145, 146). PDGF is normally expressed in a variety of cell types, including fibroblasts, neuronal cells, macrophages, smooth muscle cells, platelets, and preosteoclastic cells. They typically use autocrine or paracrine mechanisms to perform their biological functions, and Imatinib can target inhibition of PDGFR (147), can inhibit the proliferation and function of these cells, thereby reducing their promotion of VM (148). In addition, drugs targeting endothelial cells, such as Bevacizumab and Ramucirumab, can inhibit the migration and proliferation of endothelial cells(EC), thereby reducing tumor vascular density and tumor growth rate (149). Bevacizumab is the first humanized anti-VEGF neutralizing antibody approved by the FDA for the treatment of metastatic colon cancer (150), Bevacizumab treatment blocked extracellular VEGF-induced apoptosis, inhibiting EC proliferation (149). In short, targeting non-cancer cells and molecules in the tumor microenvironment can effectively inhibit tumor angiogenesis and growth, a potential tumor treatment strategy (**Table 2**).

**Table 2:** Potential drugs that inhibit vascular mimicry and their targets inhibition.

Agents	Target	Tumor type	Cell/animal	Ref.
Kaempferol	AR	Prostate cancer	HEK293/PC3/LNCaP	(59)
RES	EphA2/VE-cadherin	Prostate cancer	PC3	(61)
Chrysin	SPHK/HIF-1α	Prostate cancer	PC3	(62)
Sorafenib	VEGFR-2	Metastatic RCC	Human	(131)
Sunitinib	VEGFR-2	Metastatic RCC	Human	(151)
Bevacizumab	VEGF	Metastatic RCC	Human	(134, 152)
MMP inhibitor	ECM	Oral squamous cell carcinoma	UMSCC1	(136)
RGD peptide	ECM	–	Mice	(137)
Rapamycin	PI3K/Akt/mTOR	Gliomas	Human	(139)
Imatinib	PDGFR	Ovarian cancer	Human	(147)
Dasatinib	PDGF	ccRCC	Human	(146, 153)

## 5 Conclusion and future directions

The review on VM in urinary tumors provides in-depth new ideas and solutions for treating urinary tumors. Tumor growth and metastasis can be slowed down by targeted inhibition of tumor VM, and VM inhibitors can also be used in combination with other therapeutic methods to increase anti-cancer effects.

However, the current study faces some challenges. First, different VM targeted inhibitors may have different efficacy for different urinary tumors. Secondly, the dose and timing of VM inhibitors in clinical application and the combination regimen and sequence of combination therapy strategies require further research.

With the in-depth study of tumor VM, the efficacy and safety of treatments targeting to inhibit VM will continue to improve. Combinational use of VM targeted inhibitors with other antitumor methods is envisaged to improve the therapeutic effect further and reduce the side effects. Therefore, we can expect targeted therapy for tumor VM to play an increasingly important role in the future.

## Author contributions

XL: validation, writing, formal analysis and visualization. SL and CY reviewed the manuscript and polished the grammar. SH, SL, XZ and GZ: searched related publications. GW and JZ: supervision. All authors contributed to the article and approved the submitted version.
